# Age- and Density-Dependent Parasitism Rate and Development Time of the Generalist Egg-Parasitoid *Ooencyrtus telenomicida* (Hymenoptera: Encyrtidae) on Eggs of the Brown Marmorated Stink Bug, *Halyomorpha halys*

**DOI:** 10.3390/insects15010014

**Published:** 2023-12-28

**Authors:** Eleni I. Koutsogeorgiou, Theodoros Moysiadis, Georgios T. Fifis, Nikoloz E. Gogolashvili, Dimitrios Chatzimpalasis, Stefanos S. Andreadis

**Affiliations:** 1 Institute of Plant Breeding and Genetic Resources, Hellenic Agricultural Organization-Demeter, P.O. Box 60458, 57001 Thermi, Greece; eikoutso@agro.auth.gr (E.I.K.); moysiadis.t@unic.ac.cy (T.M.); 2 Laboratory of Applied Zoology and Parasitology, School of Agriculture, Aristotle University of Thessaloniki, 54124 Thessaloniki, Greece; 3 Department of Computer Science, School of Sciences and Engineering, University of Nicosia, Nicosia 2417, Cyprus; 4 Division of Agriculture, International Hellenic University—Sindos Campus, 57400 Sindos, Greece; gakiasfif@gmail.com (G.T.F.); gogolanick@gmail.com (N.E.G.); 5 School of Biology, Aristotle University of Thessaloniki, 54124 Thessaloniki, Greece; dimitris.hatzis.98@gmail.com

**Keywords:** brown marmorated stink bug, egg parasitoid, parasitism rate, development time, age, density, oviposition experience

## Abstract

**Simple Summary:**

The brown marmorated stink bug, *Halyomorpha halys*, is a pentatomid bug that feeds on a wide range of economically important crops. Crop protection in newly invaded areas mainly relies on the use of broad-spectrum chemical insecticides, while little to no information is available regarding a classical biological control approach. The overall purpose of the present study was to estimate the parasitism rate and development time of the native egg parasitoid *Ooencyrtus telenomicida* Vassiliev (Hymenoptera: Encyrtidae) when offered *H. halys* egg-masses, taking into consideration several parameters. Specifically, we tested different parasitoid ages and densities on 1–4-day-old *H. halys* eggs. According to our results, parasitoid density and the age of host eggs significantly affected the parasitism rate of *O. telenomicida*. High parasitoid density along with younger host eggs led to higher parasitism rates. Likewise, a significant interaction was observed between the parasitoid density and their age. Successful parasitisation occurred with host eggs up to 4 days old, with parasitism rates decreasing as host egg age increased. A three-fold higher parasitism rate of *H. halys* eggs was observed when they were parasitised by older *O. telenomicida* females, compared to younger ones. Concerning the development time of *O. telenomicida*, individuals that developed in younger host eggs displayed a shorter development time. Parasitoid age did not affect the development time of *O. telenomicida*, nor did the density of parasitoids.

**Abstract:**

*Halyomorpha halys* (Hemiptera: Pentatomidae) is an invasive pest species that was imported into Greece in 2011 and since then, has caused severe qualitative and quantitative damage to economically important crops. Its management relies mainly on the use of broad-spectrum insecticides, with little to no information available concerning the potential use of native parasitoids in terms of classical biological control. Our study aimed to assess the parasitism rate and development time of the gregarious egg parasitoid *Ooencyrtus telenomicida* (Hymenoptera: Encyrtidae) on *H. halys* egg-masses, depending on several factors such as: (i) age of parasitoids, (ii) density of parasitoids, (iii) age of host eggs, and (iv) oviposition experience of parasitoids. According to our results, the younger the host eggs and the more parasitoids, the higher the parasitism rate achieved by adults of *O. telenomicida*, with the maximum mean value of the parasitism rate observed with 1-day-old host eggs and 4 parasitoid pairs (57.3%). On the contrary, the lowest mean value of the parasitism rate was observed with 4-day-old host eggs and 1 parasitoid pair (6.5%). Similarly, the age of parasitoids significantly affected parasitisation. The older the parasitoids were, the higher the parasitism rate achieved by adults of *O. telenomicida*, with a three-fold higher parasitism rate observed at 3–4 and 5–6-day-old *O. telenomicida,* compared to 1–2-day-old (31.8, 32.4, and 12.1%, respectively). Individuals that developed in younger host eggs displayed a shorter development time, and the shortest development time was observed for *O. telenomicida* laid by 2 parasitoid pairs. Parasitoid age did not affect the development time of *O. telenomicida*, although there was a tendency for individuals laid by younger female parasitoids to exhibit a shorter development time. Our findings provide valuable information on the potential use of *O. telenomicida* as a biocontrol agent of *H. halys.*

## 1. Introduction

The brown marmorated stink bug (BMSB), *Halyomorpha halys* (Stål) (Hemiptera: Pentatomidae), is an invasive pest native to East Asia, accidentally introduced to North America and Europe [1,2]. It causes severe damage to a wide variety of important fruit trees, vegetables, industrial crops, as well as ornamental plants [1,2]. Additionally, *H. halys* is considered a nuisance pest that is likely to aggregate in man-made structures in late autumn to overwinter, and protects itself from harsh winter conditions [3,4]. In Greece, BMSB was first recorded in the fall of 2011, causing nuisance issues in houses in the centre of Athens [5]. However, a few years later in 2017, the first BMSB damage to kiwi fruits was observed in two different locations in Northern Greece [6].

The main approach against BMSB relies mainly on the use of broad-spectrum chemical insecticides that were found to be only partially effective [7,8,9], incompatible with already established IPM programs [7,9], harmful to the environment [10], and providing only a short-term solution. A potential long-term and low-cost strategy to suppress the BMSB population is the classical biological control method using natural enemies [11,12,13]. However, native natural enemies across the newly invaded areas are not considered effective for biological control due to inefficiency in significantly reducing *H. halys*’ population in most cropping systems [14,15,16,17].

Among the natural enemies that have co-evolved with *H. halys* in its native range, the samurai wasp, *Trissolcus japonicus* (Ashmead) (Hymenoptera: Scelionidae), has been identified as the most specialised and efficient agent for classical biological control of *H. halys* populations, with high parasitism rates reaching up to 70%. *Trissolcus mitsukurii* (Ashmead) (Hymenoptera: Scelionidae) is the main parasitoid in Japan [1,18,19,20]. In another study, Lara et al. [21] collected *T. japonicus* individuals from China, and tested its host range and host specificity under laboratory conditions, suggesting that it is oligophagous with a preference for BMSB as a reproductive host compared to other non-target pentatomid species. Adventive populations of *T. japonicus* and *T. mitsukurii* have been reported worldwide [12,17,22,23,24,25,26]. In Europe, among the native parasitoids that have been recorded to attack *H. halys* eggs in the field are the generalists, *Anastatus bifasciatus* and *Ooencyrtus telenomicida* [2,13,14,15]. Both species can emerge as adults from the new host [27].

As is typical of *Ooencyrtus* spp. [28], *O. telenomicida* is gregarious. The oviposition behavior of *O. telenomicida* is complex and includes drumming, drilling, and host feeding [29]. During host establishment, the female punctures the host egg with the ovipositor and feeds on the ooplasm emerging from the wound. Females repeat the drilling-host feeding sequence from 12 to 36 times before laying their eggs [30]. *Ooencyrtus telenomicida* can complete development in *H. halys* eggs [11,13] and responds to volatiles from *H. halys* males and attacked plants with a deposited egg mass in olfactometer bioassays [27]. However, it has been described as polyphagous, attacking several hemipteran and lepidopteran eggs, and can also perform as a facultative hyperparasitoid of *Eurygaster integriceps* Puton (Hemiptera: Pentatomidae) [31,32,33,34]. Thus, it is likely that host detection and establishment of this parasitoid are affected by a wider range of cues and factors. *Ooencyrtus telenomicida* has been reported to be widespread in Europe, Asia, and Africa [13,15,17,32]. There have been reports of its population being positively influenced by annual rainfall [35].

The aim of the present study is to evaluate *O. telenomicida* as a biocontrol agent against *H. halys*, by assessing its parasitism rate on BMSB egg masses depending on several factors, such as: (i) age of parasitoids, (ii) density of parasitoids, (iii) age of host eggs, and (iv) previous oviposition experience, and their possible interactions.

## 2. Materials and Methods

### 2.1. Colony Maintenance

*Halyomorpha halys* (Stål) (Hemiptera: Pentatomidae) individuals used in this study were reared on green bean (*Phaseolus vulgaris*) pods and green bean plants in mesh cages (30 × 30 × 30 cm) with a vinyl window and zip closure (Raising Butterflies, Salt Lake City, UT, USA). They were maintained at 26 °C, 60% RH, under a 16:8 h light–dark photoperiod. This colony was initiated in 2019 and originated from mixed-sex adults and nymphs that were collected from homes and fields in central Macedonia. The colony’s diet consisted of potted green bean plants and fresh fruits and vegetables (apples, kiwis, carrots, peppers, and green beans). Adult females typically laid eggs on the underside of the green bean leaves or on the top or side of the cage. Egg masses were removed and placed on the top of a small green bean leaf attached to a 4 mL clear screw vial (45 × 14.7 mm; BGB, Lörrach, Germany) that was glued to the bottom of a 460 mL round clear plastic cup (9 cm diameter × 7 cm high) (Jumbo S.A., Athens, Greece). Cotton balls soaked in water were added to the cups to increase humidity. First-instar nymphs (L_1_) were allowed to feed on fresh bean leaves. As soon as they molted into second-instar nymphs (L_2_), they were transferred in groups of 10 into new cups with 2–3 green beans. Featherweight forceps (BioQuip Products, Rancho Dominguez, CA, USA) were used to carefully handle the nymphs and avoid injury or death. Green beans were replaced two or three times per week to keep them fresh. Once nymphs reached the adult stage, they were transferred immediately into the mesh cages to minimise cannibalism. For the experimentation, egg masses were removed carefully with the help of a size 0 artist paint brush (Artist’s Loft™, MSPCI, Irving, TX, USA) daily and placed on Petri dishes (60 mm diameter), labelled by date to monitor the age of host eggs. Moistened cotton balls were added to the dishes to increase humidity.

### 2.2. Parasitoid Colony

*Ooencyrtus telenomicida* adults were obtained in the summer of 2020 from parasitised *H. halys* eggs collected from apricot trees (*Prunus armeniaca* L.) in Thermi, Thessaloniki, Northern Greece (40°32′17′′ N, 23°00′04′′ E). A colony of *O. telenomicida* was established for multiple generations at the Institute of Plant Breeding and Genetic Resources, Laboratory of Entomology at 26 °C, 60% RH, and under a 16:8 h light–dark photoperiod. Parasitoids were reared in the laboratory using fresh *H. halys* egg masses that were placed at the bottom of 460 mL round clear plastic cups. Cups were closed with a plastic lid with a 250 mm mesh net for air circulation. Adults were provided with pure honey drops ad libitum as food and moistened cotton balls as a water source, which were replenished every other day. Two egg masses were added to the parasitoid rearing cups for 48 h, after which they were removed and transferred to new cups called “incubation cups”. Adult wasps that emerged from these incubation cups were either used in experiments or returned to the rearing cups. Those used for experiments were previously sexed. Laboratory tests were conducted from October to December 2020.

### 2.3. Experimental Design

No-choice tests were designed to determine the effect of (i) age of parasitoids, (ii) number of parasitoids, (iii) age of host eggs, and (iv) previous oviposition experience of parasitoids on the parasitisation capability of *O. telenomicida* on *H. halys* egg masses.

In each experimental setup, *O. telenomicida* females of a particular age (1–2 days-old, 3–4 days-old, 5–6 days-old) and density (1 female parasitoid, 2 female parasitoids, 4 female parasitoids) were tested simultaneously on egg masses of *H. halys* (26–28 eggs) of a particular age (1-day old, 2-days old, 3-days old, 4-days old) (5 replicates per treatment, N = 5). All egg masses and parasitoids were taken from the mass-reared colony described above. Each treatment contained a single egg mass (26–28 eggs). Egg masses were placed on the lid of a plastic Petri dish (3.5 cm in diameter) and exposed to parasitoid females for 24 h in transparent plastic cylindrical vials (7.5 cm in diameter, 8.8 cm in height). A cotton wick saturated in honey water, placed on the bottom of a plastic Petri dish (3.5 cm in diameter), was provided as a food source for the parasitoid. After 24 h, the parasitoids were removed, and the eggs were reared at 26 °C, 60% RH, and under a 16:8 h light–dark photoperiod. Successful adult parasitoid emergence was recorded daily between 10 and 30 d. Treatments were replicated 5 times for each treatment. Only replicates in which parasitoid attack by *O. telenomicida* occurred on one or more eggs were included in the analysis. To obtain female parasitoids with oviposition experience, females were offered a single egg mass to oviposit 24 h prior to the experiment. For this reason, tested females with oviposition experience were 3–4 and 5–6 days old.

The following parasitism parameters were calculated: (1) parasitism rate, calculated as the number of offspring emergence over the total number of tested eggs; (2) development time of the parasitoid, calculated as the number of days from the input day until parasitoid emergence.

### 2.4. Statistical Analysis

The statistical analysis had a twofold aim. First, to assess the impact of (i) age of parasitoids, (ii) number of parasitoids, (iii) age of host eggs, and (iv) previous oviposition experience on the parasitism rate of *O. telenomicida*. Subsequently, the same four factors were assessed regarding the development time. To assess these hypotheses, the generalised linear mixed-effects model (GLMM) and the linear mixed-effects model (LMM) were employed, depending on the nature of the response variable. In particular, when the response was represented by the parasitism rate (proportion), the GLMM was used with the binomial family of distributions and the logit link, employing the function “glmer” in R [36]. To assess the presence of over/underdispersion, the R function “testDispersion”, which performs simulation-based tests, was employed within the DHARMa R package [37]. In the case that the response was represented by the development time (continuous), the LMM was used by applying the R function “lmer” [36]. The variable age of parasitoids (AoP), number of parasitoids (NoP), age of host eggs (AoE), and previous oviposition experience (OE) were included in the model as fixed effects. Replication was included as a random effect.

In both response cases, a similar methodology was applied, including two steps. Within the first step, the corresponding model was applied in three different scenarios, assessing the impact on the response of (a) AoE and NoP, (b) AoE and AoP, and (c) AoP and NoP. In all scenarios, the R function “emmeans” was then employed to compute the estimated marginal means (EMMs) for the dependent variables, perform posthoc comparisons, and visualise the corresponding results [38]. The *p*-values, in the post hoc analysis, were adjusted for multiple comparisons, based on the Bonferroni correction. Next, the complete model was applied, including all four variables (AoP, NoP, AoE, and OE). The significance level was set at 5%. All the analyses were performed with the statistical computing software R v.4.2.1 [39].

## 3. Results

**Figure 1:**
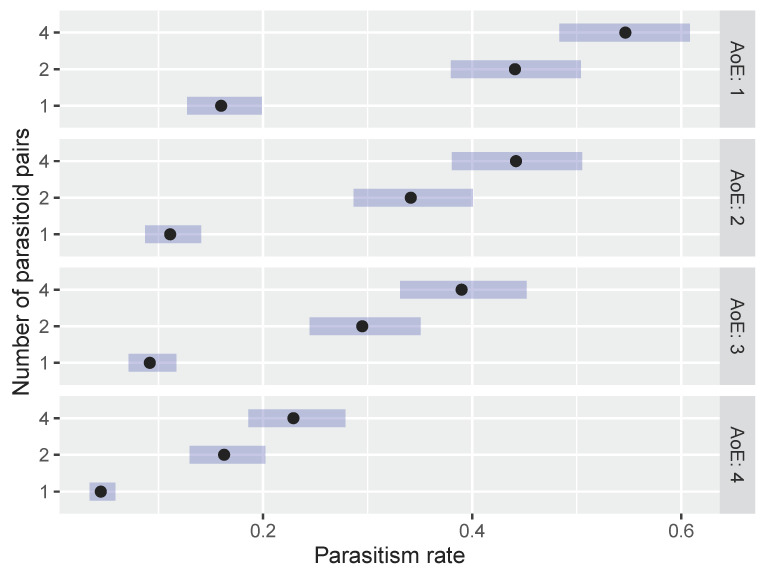
Effect of the number of parasitoid pairs on the parasitism rate of *O. telenomicida* on different ages of *H. halys* eggs. AoE stands for the age of *H. halys* eggs. The estimated marginal means (EMMs) are displayed for the parasitism rate of *O. telenomicida* (black dots) along with their 95% confidence intervals (rectangles).

Before fitting the models, the existence of over/underdispersion was assessed, implying the presence of only small to moderate overdispersion. The parasitism rate of *O. telenomicida*, when studied within the first scenario (AoE and NoP), exhibited higher values (EMMs) with the treatment of 4 pairs of parasitoids at each tested age of host eggs, followed by 2 and 1 pair of parasitoids. The highest parasitism rate was observed for 4 pairs and AoE 1 (0.547, 95% CI: 0.483–0.608) (Figure 1). This difference was found to be statistically significant compared to all other combinations of AoE and NoP (Excel S1). On the other hand, 1 pair of parasitoids and AoE 4 exhibited the lowest parasitism rate among all treatments (0.045, 95% CI: 0.034–0.059). Statistically significant differences in the parasitism rate were observed between 4 and 2 pairs of *O. telenomicida* at every age of host eggs (Excel S1).

**Figure 2:**
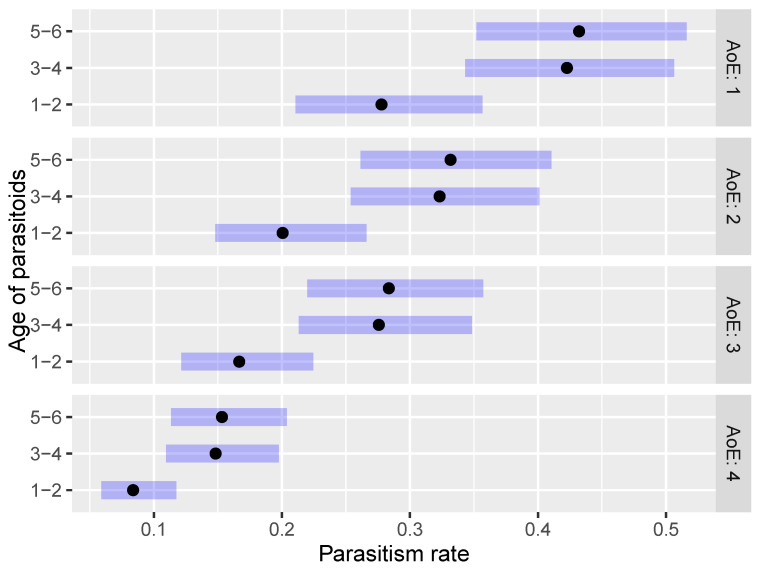
Effect of the age of parasitoids on the parasitism rate of *O. telenomicida* at different ages of *H. halys* eggs. AoE stands for the age of *H. halys* eggs. The estimated marginal means (EMMs) are displayed for the parasitism rate of *O. telenomicida* (black dots) along with their 95% confidence intervals (rectangles).

A similar pattern was observed within the second scenario (AoE and AoP), i.e., the older the parasitoids, the higher the parasitism rate (EMM) observed at each tested age of host eggs. More specifically, higher parasitism rates were observed for the 5–6 days-old parasitoids at each tested age of host eggs, followed by 3–4 days-old and 1–2 days-old parasitoids (Figure 2). There was no statistically significant difference in the parasitism rate between 5–6 days-old and 3–4 days-old *O. telenomicida* at each tested age of host eggs (Figure 2 and Excel S2). On the other hand, the parasitism rate of 1–2 days-old *O. telenomicida* was significantly lower compared to both 5–6 days-old and 3–4 days-old pairs at each tested age of host eggs (Figure 2 and Excel S2).

**Figure 3:**
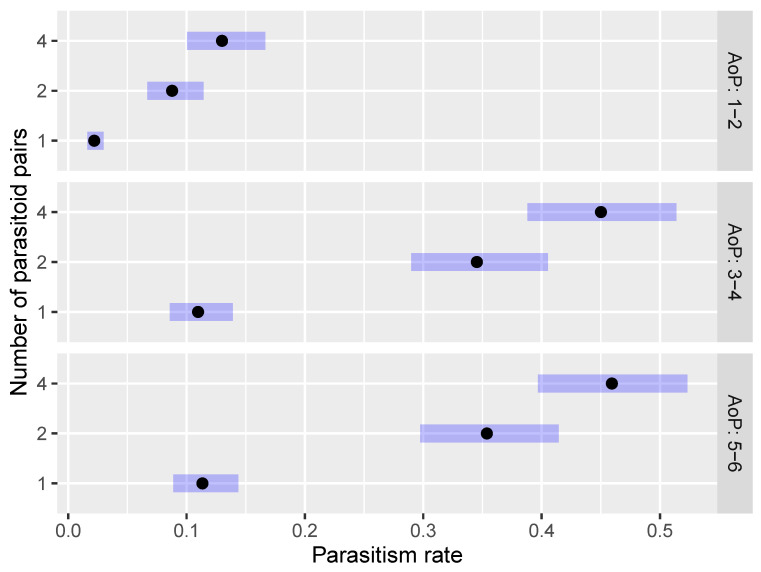
Effect of the number of parasitoid pairs on the parasitism rate of *O. telenomicida* at different ages of adult parasitoids. AoP stands for the age of *O. telenomicida* adults. The estimated marginal means (EMMs) are displayed for the parasitism rate of *O. telenomicida* (black dots) along with their 95% confidence intervals (rectangles).

The parasitism rate (EMM) was higher when H. halys eggs were treated with 4 pairs of *O. telenomicida*, regardless of the age of parasitoids, followed by 2 and 1 pair of parasitoids (Figure 3). There was a statistically significant difference between the parasitism rate of 4 pairs and 2 pairs of *O. telenomicida*, as well as between 2 pairs and 1 pair (Figure 3). The parasitism rate of all tested number of pairs of parasitoids significantly declined with the decreasing age of parasitoids (Figure 3 and Excel S3). The highest parasitism rate was observed for 4 pairs and AoP 5–6 (0.459, 95% CI: 0.397–0.523) (Figure 3), and the lowest for 1 pair and AoP 1–2 (0.022, 95% CI: 0.016–0.030) (Figure 3).

The parasitism rate of *O. telenomicida* across different categories of the number of parasitoids, the age of host eggs, and the previous oviposition experience is displayed in Figure S1. The highest values for each egg age are observed when the NoP equals 4 and without OE, with the exception of AoE 3. Similarly, the development time of *O. telenomicida* across different categories of the abovementioned factors is displayed in Figure S2. The highest values for each egg age are observed when the NoP equals 1 and without OE, with the exception of AoE 4.

In Table 1, the results of the complete model (including all four variables AoP, NoP, AoE, and OE) concerning the parasitism rate of *O. telenomicida* are displayed. Specifically, the parasitism rate was significantly affected by the number of parasitoids, the age of parasitoids, the age of host eggs, and the previous oviposition experience (NoP: χ^2^ = 614.33, d.f. = 2, *p* < 0.001; AoP: χ^2^ = 288.52, d.f. = 2, *p* < 0.001; AoE: χ^2^ = 307.28, d.f. = 3, *p* < 0.001; OE: χ^2^ = 21.82, d.f. = 1, *p* < 0.001) (Table 1).

**Figure 4:**
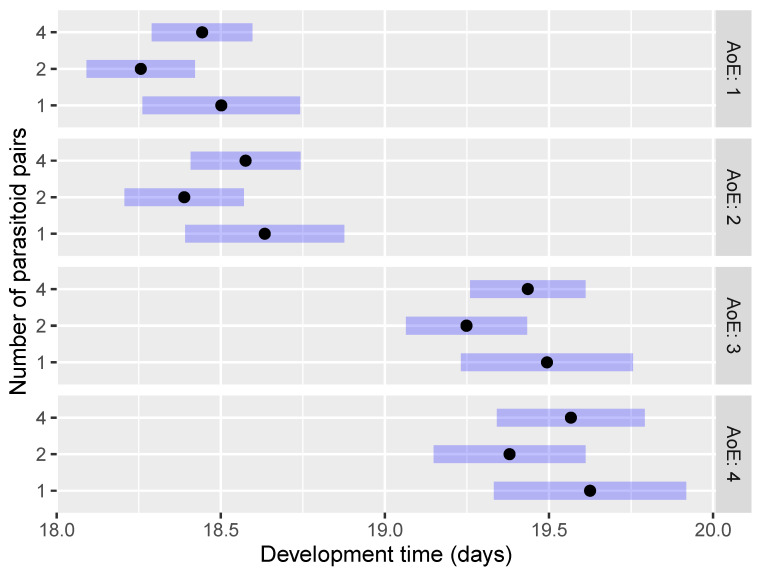
Effect of the number of parasitoid pairs on the development time of *O. telenomicida* at different ages of *H. halys* eggs. AoE stands for the age *H. halys* eggs. The estimated marginal means (EMMs) are displayed for the development time (days) of *O. telenomicida* (black dots) along with their 95% confidence intervals (rectangles).

The development time (EMMs) of *O. telenomicida* within the first scenario (AoE and NoP) showed no statistically significant differences in the pairwise comparisons concerning the number of parasitoid pairs at each age of host eggs (Figure 4 and Excel S4). The only exception was the comparison NoP2 AoE2—NoP2 AoE4 (*p* < 0.001). The shortest development time was observed for the treatment of 2 pairs of *O. telenomicida* at each tested age of host eggs followed by 1 and 4 pairs of parasitoids (Figure 4).

**Figure 5:**
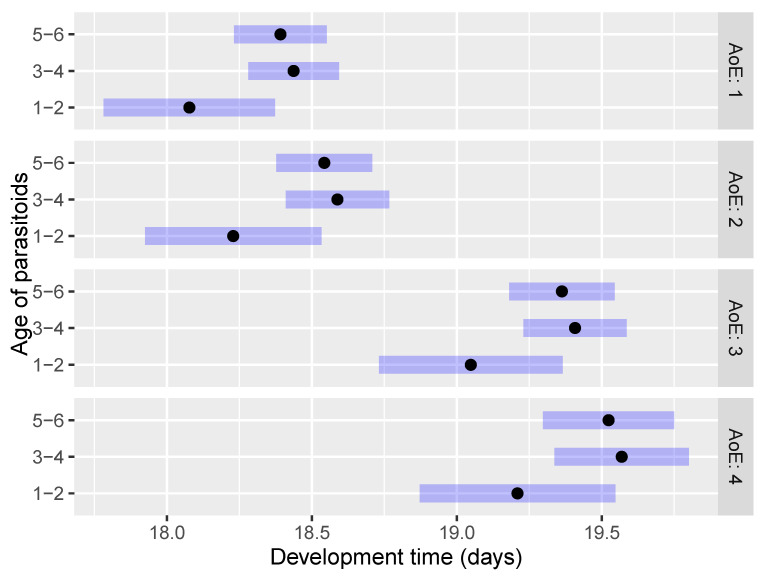
Effect of the age of parasitoids on the development time of *O. telenomicida* at different ages of *H. halys* eggs. AoE stands for the age of *H. halys* eggs. The estimated marginal means (EMMs) are displayed for the development time (days) of *O. telenomicida* (black dots) along with their 95% confidence intervals (rectangles).

Furthermore, the development time of *O. telenomicida* was significantly shorter on 1 and 2 days-old host eggs compared to 3 and 4 days-old eggs, regardless of the number of parasitoid pairs (Figure 4) and the age of the parasitoids (Figure 5 and Excel S5). Our results suggest that the age of parasitoids did not affect *O. telenomicida’* development time. Although offspring of 1–2 days-old parasitoids displayed a shorter development time compared to that of older ones at each tested age of host eggs, this difference was not statistically significant (Figure 5).

**Figure 6:**
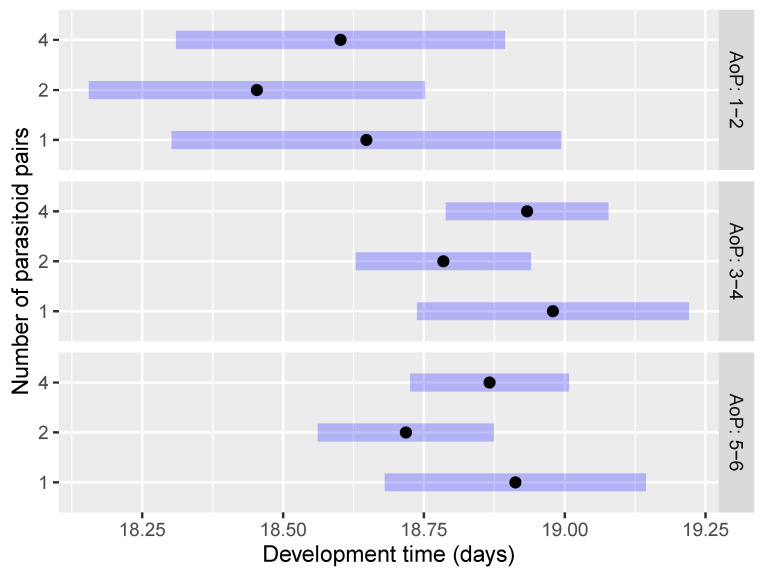
Effect of the number of parasitoid pairs on the development time of *O. telenomicida* at different ages of adult parasitoids. AoP stands for the age of *O. telenomicida* adults. The estimated marginal means (EMMs) are displayed for the development time (days) of *O. telenomicida* (black dots) along with their 95% confidence intervals (rectangles).

The shortest development time was observed for the treatment of 2 pairs of *O. telenomicida* at each tested age of parasitoids, followed by 1 and 4 pairs of parasitoids (Figure 6), but no statistically significant differences were observed among treatments with a different number of pairs of *O. telenomicida* at each tested age of parasitoids (Excel S6).

The results of the complete model (including all four variables AoP, NoP, AoE, and OE) concerning the development time of *O. telenomicida* are shown in Table 2. Our results show that the development time was significantly affected only by the age of host eggs (AoE: χ^2^ = 192.20, d.f. = 3, *p* < 0.001) (Table 2).

## 4. Discussion

According to our results, the younger the host eggs were and the more parasitoids attacking them, the higher the parasitism rate observed. As host eggs got older, successful parasitisation declined. On the contrary, the older the parasitoids were, the higher the parasitism rate observed, regardless of the host egg age. This is in agreement with a study by Power et al. [40], where they showcased that the best combination for high parasitisation of the pentatomid *B. hilaris* eggs by *O. mirus* was 0–1-day-old host eggs with 3–10- day-old parasitoids. The least successful host egg age was the oldest, and the least successful parasitoid age was the youngest. Our findings also agree with Tunca et al. [41], who evaluated the parasitism rate of *Ooencyrtus pityocampae* Mercet (Hymenoptera: Encyrtidae) on eggs of *Philosamia ricini* Hutt. (Lepidoptera: Saturniidae) and found that the optimal age of host eggs for parasitisation was 1–2 days. The same study stated that older parasitoids laid more eggs, making 5-day-old parasitoids the most promising age for the highest emergence rate. Indeed, it has been previously discussed that host eggs older than 4 days are generally less suitable for *Ooencyrtus* species, although they may be accepted for parasitoid oviposition [28,42,43]. This may be explained by the changes in the chemical content and the properties of insect eggs taking place over time which affect the suitability of the host for successful development of the parasitoid. Host age appeared to significantly affect the feeding, resting, and oviposition behavior of *Ooencyrtus nezarae* Ishii (Hymenoptera; Encyrtidae) on *Riptortus clavitus* (Thunberg) (Hemiptera: Alydidae) eggs [38]. According to their results, although all host ages were accepted by the parasitoids, the number of feeding bouts and eggs laid per host decreased as host age increased, and parasitoids spent less time feeding or resting on older host eggs than they did on 0-day-old ones. Similar results were discussed by Hofstetter and Raffa [43], who investigated the parasitism rate of *Ooencyrtus kuvanae* (Howard) (Hymenoptera: Encyrtidae) on eggs of *Lymantria dispar* (L.) (Lepidoptera: Erebidae). Relatively fewer parasitoid individuals emerged from older egg masses, and parasitoid reproduction decreased with host egg age.

Our study adds to the notion that a younger age of the host seems more appropriate for the development of offspring by egg parasitoids. Indeed, several studies with egg parasitoids, including *Telenomus heliothidis* Ashmead, *Telenomus solitus* Johnson, *Telenomus remus* Nixon, and *Triphodytes gerriphagus* Marchal (Hymenoptera: Scelionidae) [44,45,46,47], highlight the low emergence rate of parasitoids reared on old host eggs and attribute it to the advanced state of embryonic development of the host. Developed stages may prevent feeding by the parasitoid larva because of the incapability of cuticle digestion.

On the other hand, the age of the parasitoids could also affect their ability to parasitise their hosts [48,49,50]. Our results show that the parasitism rate increased with parasitoid age and number. Tunca et al. [51] evaluated the potential of *O. kuvanae* as a biocontrol agent for *H. halys* and observed that the highest parasitism rate occurred by 5 and 7-day-old parasitoids. In a study by Aung et al. [52], the percentage parasitism and the number of progeny of *O. nezarae* when parasitising eggs of *R. clavatus* were significantly decreased in old females (20 days old). The optimal age of *O. nezarae* for successful parasitism ranged from 1 to 4 days [52]. In our study, the younger the female parasitoids we used in our treatments, the lower the percentage of successful parasitisation we observed. Likewise, parasitoid age (1 to 5 days old) of *Trichogramma pretiosum* (Riley) (Hymenoptera: Trichogrammatidae) significantly affected its efficacy on *Anticarsia gemmatalis* (Huebner) (Lepidoptera: Erebidae) eggs. A higher number of parasitoids per egg emerged after parasitism by older *T. pretiosum* females (5 days old) [53].

Moreover, the density of parasitoids significantly affected the parasitism rate of *O. telenomicida.* The higher the density of female parasitoids was, the higher the percentage of successful parasitisation we observed. Likewise, Feliciangeli and Rabinovich [53] observed that the percentage of parasitism increases rapidly with the parasite density of *Ooencyrtus trinidadensis* Crawford (Hymenoptera: Encyrtidae); however, there is a maximum in the number of hosts parasitised per female that shifts towards larger numbers of host eggs available with increasing parasite density, reflecting a strong interaction between parasite and host densities [54].

The development time of the immature stage of parasitoids may vary with the size, age, and quality of the host in which they were reared [55]. As a matter of fact, our results show that the host age of *H. halys* does not only affect the parasitism rate but also the development time of immature *O. telenomicida*. The younger the host eggs, the shorter the development time of *O. telenomicida*. This particular observation is in accordance with previous reports on related *Ooencyrtus* species, such as *O. mirus.* The development time of *O. mirus* on 0–4-day-old *B. hilaris* eggs was significantly shorter than in 5-day-old host eggs [40]. On the other hand, parasitoid offsprings laid by 0-day-old *O. mirus* females took significantly more time to develop than those laid by older female parasitoids [40]. This also agrees with our finding that the younger the parasitoids, the shortest the development time, although no statistically significant difference was observed among treatments. Young hosts ensure that the necessary development time is available for the successful development of parasitoid offspring and also provide the best host quality [56].

In contrast with the observations conducted in the present work related to the previous oviposition experience of *O. telenomicida* females, Wang et al. [57] reported that oviposition experience influenced the female reproductive behaviour of *Anastatus japonicus* Ashmead (Hymenoptera: Eupelmidae). When an egg clutch of *Antheraea pernyi* (Guerin-Meneville) (Lepidoptera: Saturniidae) was offered for oviposition, females with oviposition experience produced significantly more offspring than that produced by females without any oviposition experience [57]. However, this is not the case in our study, where oviposition experience neither affected the parasitism rate nor the development time of *O. telenomicida*, regardless of the treatment we tested.

## 5. Conclusions

Our study showcases the combined effect of host egg age, parasitoid age, and parasitoid density on the parasitism rate of *O. telenomicida* when parasitising eggs of *H. halys.* Our results suggest that the most successful parasitisation occurs when the host eggs are younger and the parasitoids are older. This may be explained by the developed embryonic state of the host eggs, which may prevent the feeding of the parasitoid due to the incapability of cuticle digestion. Our study also explores the density of the parasitoids that is most suitable for high parasitism rates, indicating that the more parasitoids attacking *H. halys* eggs, the higher the parasitism rate. However, there seems to be an interaction between host density and parasitoid density, influencing parasitism rates, as observed in previous studies [54]. In conclusion, our study provides valuable insight into the different factors affecting *H. halys* egg parasitisation and how these interact with each other, adding to a better understanding of potential biocontrol agents for this insect and their incorporation into biological management programs.

## Figures and Tables

**Table 1:** Results after applying the generalised linear mixed-effects model, and the linear mixed-effects model to assess the impact of the number of parasitoid pairs (NoPs), age of parasitoids (AoP), age of host eggs (AoE), and previous oviposition experience (OE) on the parasitism rate (number of emerged adult parasitoids over the total number of tested eggs).

	Chisq	d.f.	*p*-Value
(Intercept)	954.06	1	<0.001
NoP	614.33	2	<0.001
AoP	288.52	2	<0.001
AoE	307.28	3	<0.001
OE	21.82	1	<0.001

**Table 2:** Results after applying the generalised linear mixed-effects model and the linear mixed-effects model to assess the impact of the number of parasitoid pairs (NoPs), age of parasitoids (AoP), age of host eggs (AoE), and previous oviposition experience (OE) on the development time of *O. telenomicida* (days since the input day until adult parasitoid emergence).

	Chisq	d.f.	*p*-Value
(Intercept)	47,728.81	1	<0.001
NoP	3.41	2	0.117
AoP	1.75	2	0.417
AoE	192.20	3	<0.001
OE	0.14	1	0.707

## Data Availability

The data presented in this study are available within the article and Supplementary Materials.

## Supplementary Materials

The following supporting information can be downloaded at: https://www.mdpi.com/article/10.3390/insects15010014/s1, Figure S1: Effect of previous oviposition experience on the parasitism rate of *O. telenomicida*; Figure S2: Effect of previous oviposition experience on the development time of *O. telenomicida*; Excel S1: Posthoc analysis for Figure 1; Excel S2: Posthoc analysis for Figure 2; Excel S3: Posthoc analysis for Figure 3; Excel S4: Posthoc analysis for Figure 4; Excel S5: Posthoc analysis for Figure 5; Excel S6: Posthoc analysis for Figure 6.
