# Supplementary figures and images for: Age- and Density-Dependent Parasitism Rate and Development Time of the Generalist Egg-Parasitoid Ooencyrtus telenomicida (Hymenoptera: Encyrtidae) on Eggs of the Brown Marmorated Stink Bug, Halyomorpha halys
Source: Insects. 2023 Dec 28;15(1):14. doi: 10.3390/insects15010014 (PMC10817064; doi:10.3390/insects15010014)

NoP   ●   1   ▲   2   ■   4   OE   ●   N   ●   Y

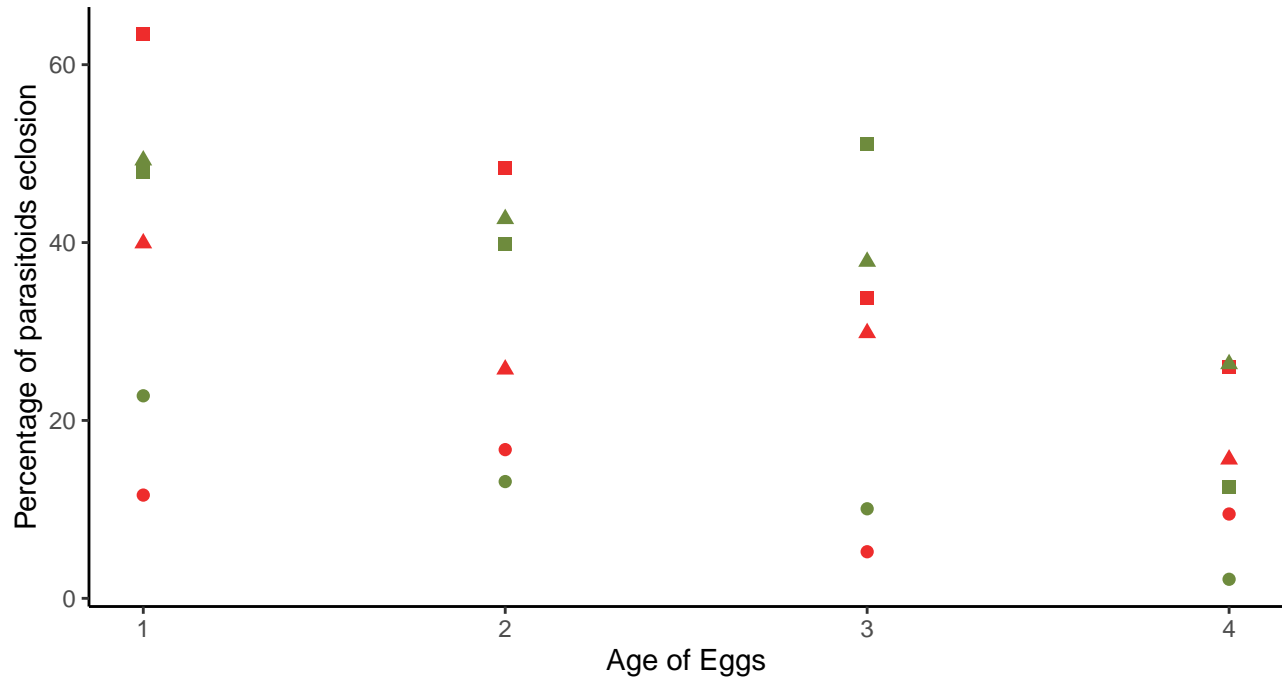

Supplement: Supplementary file 1 [file insects-15-00014-s001.zip › Figure S1.pdf]

NoP ● 1 ▲ 2 ■ 4 OE ● N ● Y

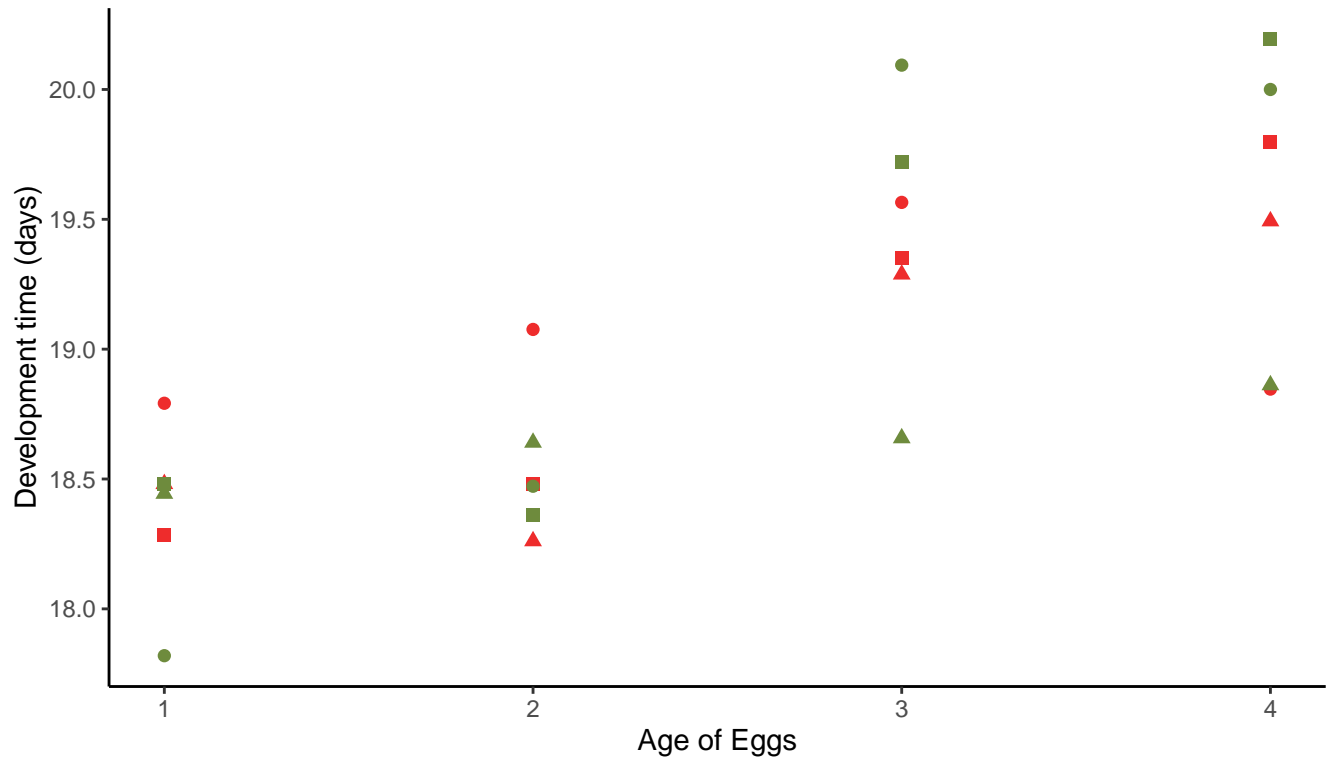

Supplement: Supplementary file 1 [file insects-15-00014-s001.zip › Figure S2.pdf]
